# Codesigning the South Asian Diet and Activity Intervention (SADAI): process and outcomes

**DOI:** 10.1017/S1368980025100839

**Published:** 2025-08-27

**Authors:** Sherly Mathew Parackal, Sivamanoj Yadav Boyina, Rachel Brown

**Affiliations:** 1 Centre for International Health, University of Otago, Dunedin, New Zealand; 2 Department of Human Nutrition, University of Otago, Dunedin, New Zealand

**Keywords:** South Asians, Diet-related NCD, Intervention development, Codesign, Coproduce

## Abstract

**Objective:**

This study outlines the development of a codesigned, coproduced intervention to address the high risk of diet-related non-communicable diseases (NCD) among South Asians (SA) in New Zealand. The objectives were to identify: (1) reasons, concerns and perceptions influencing dietary changes post-migration; (2) preferred formats and delivery modes for the intervention; (3) intervention design features; (4) community volunteers for coproduction; and (5) coproduce the intervention components.

**Design:**

Participatory Action Research.

**Setting:**

SA communities in Auckland and Dunedin, New Zealand.

**Participants:**

SA immigrants aged 25–59 years. Ten telephone or face-to-face interviews were conducted between 2018 and 2019. Following this, one codesign workshop (*n* 12) was conducted with the target population and community stakeholders in 2019.

**Results:**

Thematic analysis revealed factors such as children’s preference for boxed cereals and limited time for traditional breakfasts contributed to poor dietary habits. Concerns included meal timing and long-term weight gain, while perceptions such as all home-cooked food is healthy, influenced a lack of concern for long-term health. Preferred formats were educational comics and video clips, with digital platforms as the delivery mode. The workshop helped choose comic characters and identify community members to coproduce video content. The final intervention included eleven comics, eight videos, twelve audio clips and eighteen scientific snippets, organised into five dietary and one physical activity module.

**Conclusions:**

A participatory approach proved feasible for codesigning a culturally tailored lifestyle intervention to address diet-NCD risks in the SA diaspora in New Zealand.

In New Zealand (NZ), South Asians (SA), that is, people from Afghanistan, Bangladesh, India, Nepal, Pakistan and Sri Lanka^(1)^, make up about 6 % of the population^(2)^. SA have a higher risk and prevalence of diet-related non-communicable diseases (diet-NCD) such as diabetes and CVD both in NZ^(3,4)^ and other migrant receiving countries such as the USA, Canada and the United Kingdom^(5–9)^. Lifestyle interventions have been consistently shown to prevent or delay diet-NCD such as diabetes and CVD. For example, lifestyle interventions have been shown to reduce the onset of type 2 diabetes among those with prediabetes^(10–14)^ decreasing the progression to diabetes by 50 %, with effects lasting for over 10 years^(12,13)^ and resulting in substantial health care cost savings^(15)^. There have been attempts made to culturally tailor interventions developed for other population groups, such as Caucasians, to address the high risk of diabetes and CVD among SA migrants. Examples of these include the Prevention of Diabetes and Obesity in SA (PODOSA) trial in Scotland^(16)^, and the SA Healthy Lifestyle Intervention (SAHELI) trial in the USA^(17)^. However, the long-term impact of such interventions has been limited, as observed in the PODOSA trial, where the intervention group experienced a mean weight loss of 1·64 kg, compared to a mean weight gain of 0·51 kg in the control group over seven years^(18)^.

Participatory Action Research (PAR) is a collaborative, iterative, open-ended approach with community relationship building at the core of this process. PAR is driven by the imperative to generate knowledge-for-action and knowledge-through-action, in meeting the needs or addressing issues faced by specific communities^(19)^. PAR recognises the need for the targeted community to fully participate in all aspects and phases of the research and have the power and control to identify solutions that are sustainable^(20)^. Such a shared decision-making approach in developing targeted interventions to address health issues has been shown to be more beneficial than one that is researcher-driven, especially among disadvantaged populations^(21)^. Minority groups in developed countries are one such marginalised and disadvantaged population and the uptake of universal interventions are poorer in such populations in comparison to majority populations, primarily due to cultural inappropriateness of the intervention^(22)^. Even culturally tailored interventions are found to be less impactful in addressing diet-NCD such as diabetes and CVD in this population^(18)^. Hence, PAR is likely the most suitable approach to develop interventions to address the health needs of minority populations.

Codesign methodology, a PAR approach, is founded on the principle that the community of interest is the expert and involves their active contribution to the design of the intervention^(23)^. Codesign is drawn from design sciences and has the distinctive feature of facilitating direct user (population of interest) and provider (researchers) face-to-face collaboration in codesigning services (intervention)^(24)^. This approach has been successfully used to develop interventions for diabetes self-management^(25)^, achieving a high attendance and completion rate (93 %)^(26)^, as well as in primary prevention of diabetes^(27)^. The aim of the study is to describe the development of an innovative codesigned and coproduced intervention to address the high risk of diet-NCD in SA living in NZ. Specific objectives were to identify (1) reasons, concerns and perceptions influencing dietary changes post-migration; (2) preferred formats and delivery modes for the intervention; (3) intervention design features; (4) community volunteers for coproduction; and (5) coproduce the intervention components

## Methods

Since 2010, a suite of formative studies aimed at prolonging the healthy migrant status of SA migrants in NZ has enabled the formation of a strong community partnership through engagement with the SA community in 2017^(28)^. This partnership enabled the formation of a SA community stakeholder group, representing the major SA countries (India, Pakistan, Bangladesh, Sri Lanka and Nepal) and religions (Hindu, Christian and Muslim) in NZ. This partnership paved the way for moving into codesigning and developing a health-promoting intervention to address unhealthy dietary and activity behaviours. This process was iterative involving two sequential studies conducted in 2018 and 2019. We have previously reported on identifying food habits that changed post-migration^(29)^, barriers and enablers of detrimental dietary and activity behaviours and generating intervention components (solutions) for addressing the identified barriers^(30)^. In this manuscript, we report findings of the semi-structured interview data that informed the intervention development and delivery as well as the codesigning and coproducing of the intervention. The interviews were audio-recorded and the codesign workshop was video-recorded and conducted by researchers of SA ethnicity to ensure that the research process was done in a culturally appropriate manner. Participants provided informed consent and completed a short demographic questionnaire prior to participation in the interviews and codesign workshop. All procedures performed in this study were in accordance with the ethical standards of the institutional research committee and with the 1964 Helsinki declaration and its later amendments or comparable ethical standards. Ethics approval was obtained from the University of Otago Human Ethics Committee (18/205; dated 14/12/2018).

### Positionality statements

Authors SMP and SYB are of South Asian ethnicity. SMP actively participated in the coproduction of three video clips and all accompanying audio clips. Author RB is a non-Indigenous researcher of European descent, living and working in Aotearoa New Zealand. Their position informs a commitment to working respectfully with diverse communities and maintaining cultural sensitivity throughout the research process.

### Study participants and recruitment

The participants for this study were first generation SA^(1)^, aged 25–59 years, who understood and could communicate in English and were living in Dunedin or Auckland, NZ. In addition, for the semi-structured interviews, participants who were early migrants (less than or equal to 5 years in NZ) were targeted as previous research has shown that the first five years is the optimal period for a health-promoting intervention^(31)^. Nevertheless, for reasons similar to the focus group discussion^(30)^, which was to capture the experiences of immigrants of different durations of residence on changing dietary habits and physical activity patterns post-migration and their consequential impact on long-term health, this criterion was not used to recruit participants for the codesign workshop. Participants meeting the study’s inclusion criteria were recruited via advertising through established links of the stakeholders and researchers to various SA cultural and religious groups in NZ. Data for this study were collected between 2018 and 2019. Members of the SA community stakeholder group were also invited to participate in the codesign workshop.

### Study design, data collection and analysis

**Fig. 1:**
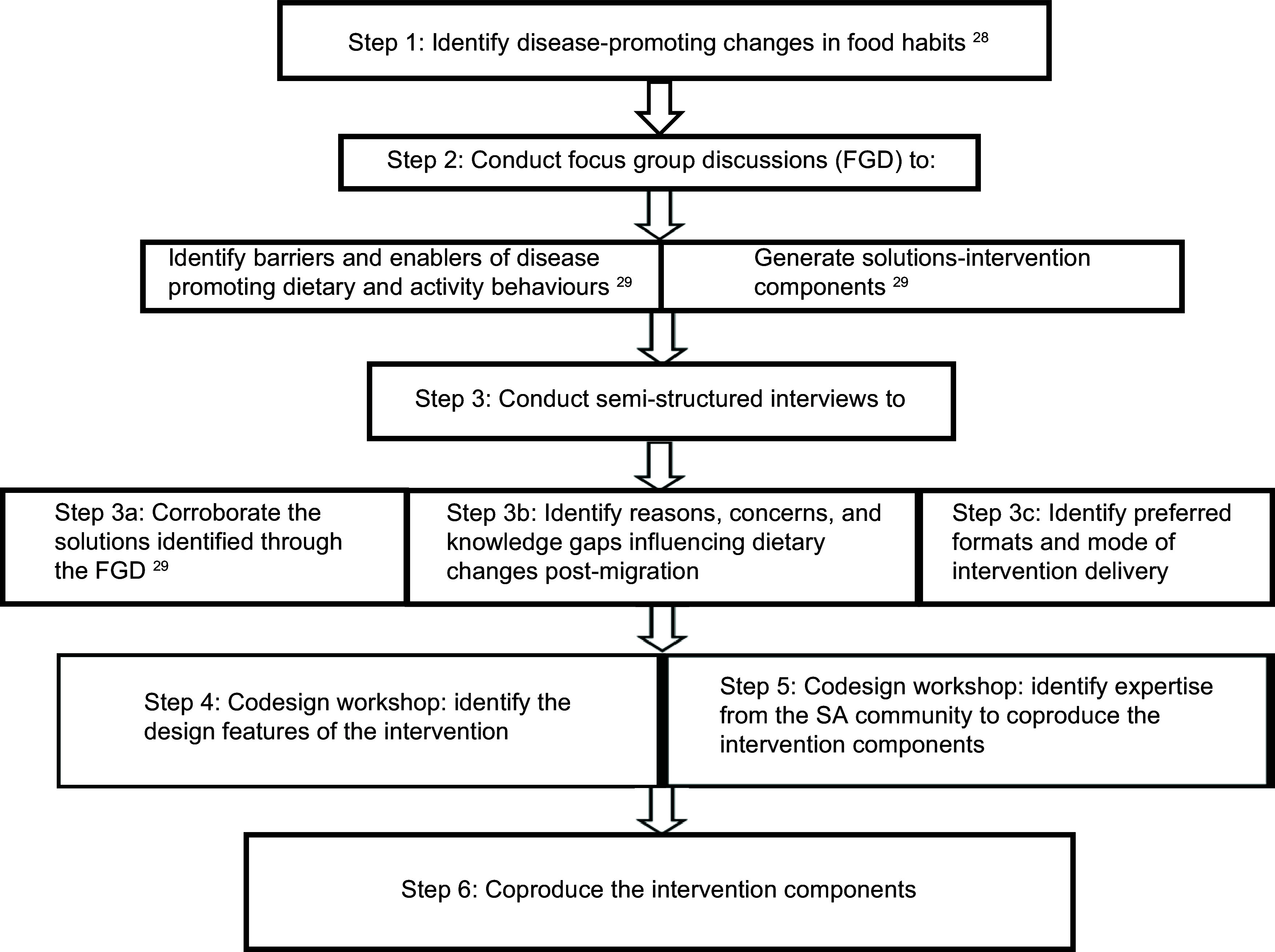
Methodology of developing the South Asian Diet and Activity Intervention.

This study used an iterative, six-step process to develop the intervention (Fig. 1). The procedures used for steps 1, 2 and 3a have been previously reported^(29,30)^. The procedures used for the thematic analysis of the semi-structured interviews have also been previously reported^(30)^. In brief, ten semi-structured interviews (45–60 min) were conducted face-to-face and via telephone. Authors SYB and SMP transcribed all the recordings verbatim, and the transcribed data were thematically analysed^(32)^ The findings of the analysis were discussed with author RB, and an agreement was reached on the behaviours and knowledge gaps to be targeted, and the preferred formats and mode of intervention delivery.

Following this, a codesign workshop (12 participants; 120 min) was conducted with the SA community (participants and stakeholders) to identify the design features of the intervention and expertise from the community to coproduce the intervention (steps 4 and 5). To ensure adherence to SA cultural norms and to foster interaction among community stakeholders, participants and investigators, the workshop was structured to begin with a buffet lunch comprising of SA cuisine. Following this, author SMP provided an overview of the target detrimental behaviours and identified solutions, and the preferred formats and mode of intervention delivery, which was followed by an open discussion to set the context for the codesign workshop.

Following this, the workshop participants were divided into three groups and asked to identify culturally appropriate design features of the intervention including the settings, scenarios and characters for developing the comics. Next, a discussion was held to identify expertise within the SA community for coproducing informational and demonstrational video clips.

### Coproduction of the intervention components

Using the characters, settings and scenarios identified through the codesign workshop, SMP developed the content and story board of eleven comics. A professional cartoonist was engaged to create the digitised education comics from the storyboards. The eleven comics were then assessed by the SA community members (*n* 5; step 6) using a 5-point Likert scale for ease of understanding the embedded health messages and the cultural appropriateness of the storyline.

Community members with expertise for producing video clips were approached by SMP and those who agreed to participate were informed of the purpose and duration of the video clips (no more than 10 min; step 6).

## Results

The findings of steps 1–3a have already been reported including the demographic characteristics of the interview participants^(30)^. The mean age of the interview participants was 33·6 (s d 3·6; *n* 10) years. The majority were women (*n* 6), from India (*n* 6), affiliated with the Hindu religion (*n* 7), employed (*n* 8), completed a tertiary education (*n* 9), had a household income between NZ $ 20 000–50 000 (*n* 6) and were early migrants who had lived in NZ for five years or less (*n* 9).

### Semi-structured interviews

#### Theme 1: reasons for changes in diet post-migration

All participants indicated their dietary habits changed post-migration. This was particularly true for breakfast items, and many participants indicated that they ate boxed cereals popular with their children for breakfast, and others reported a lack of time to prepare traditional breakfast items as a reason for post-migration changes in breakfast habits.

*Because I buy the Chocos for my kids, and sometimes I also eat that… **Participant 8***

*I think if you compare when I was back home, we used to have some type of breakfast, like dosa or idli, (traditional breakfast items made from rice and lentils) I mean sometype (of dals(lentils)). Everyday you’ll have a breakfast which has the dals and lentils. But, after coming here, I was not having idli or dosa. **Participant 7***

*It’s like, time frame. It takes little (some) time for me to wake up early in the morning and prepare. dosa and idli (traditional breakfast items made from rice and lentils) has become only a (an) occasional food, just once or twice in a month, for me. **Participant 2***

#### Theme 2: concerns about the impact of post-migration changes in diet on health

Meal timing was a concern for some participants who reported that eating was dependent on their work schedule.

*If I am at home, if I am on a day off, I’ll not be hungry, even till 12:00 PM. For some people, they wanted breakfast, immediately, after wake up. They wanted to eat something and all, but I don’t know. Maybe for me, I’ll not feel hungry, if I don’t go to work. If I go to work, then definitely, by 11:00 AM or 12:00, I’ll be hungry. I wanted something to eat then. **Participant 2***

Seven out of ten participants feared their current dietary habits could have negative health impacts in the long term.

*I’m a little bit concerned because we do have a diabetic history. That’s the reason, but if there was no diabetic history, I guess I wouldn’t be so concerned about that. **Participant 3***

*Yeah, I would say (I am concerned) because I think after six months I started gaining a little bit of weight, because I think I was having a lot of sugary foods here after moving to NZ. so, yeah, maybe because there is a lot of excess of sugary foods escalating like that. A lot of ice-creams**. Participant 1***

*I’m really afraid to be honest (of impact on health), yes. I’m just concerned. **Participant 5***

*I thought like if I would continue doing this (eating unhealthy foods),…my obesity would grow and I would become fat maybe. **Participant 9***

Perceptions such as home-cooked foods are ‘automatically’ healthy and limited awareness of the long-term impact of poor dietary habits, contributed to a lack of concern about the impact of poor diet on future health.

*No. No concern as such because I only eat home food. I don’t need (eat) outside and more because I’m a vegetarian, so I have hardly any options (to eat) outside. I mean, because I know a bit of science, I do try to make (healthy) food. **Participant 4***

*I’m fortunate enough, I eat similar food, what I used to have in India. Reason is, my wife cooks very nice Indian food and I prefer Indian food… my wife cooks pretty much everything. **Participant 6***

*Yeah, I’m not super concerned about, in the future (maybe), but right now, I’m just trying to be (survive), whatever I get, I want to eat, at the moment. I will make sure, not to eat so much of unwanted food which will cause a trouble in the future. **Participant 2***

#### Theme 3: preferred formats for delivering the health messages

Participants reported a preference for health messages to be presented visually such as video clips in contrast to information they have to read and understand.

*. when you put it (health messages) in a (on) YouTube and it’s visual, it attracts (your attention) immediately. Short informative videos not more than two, three minutes, maximum five minutes, … If they see it’s one or two minute and with the very good title or caption on it, as long as it has a very good immediate attraction on it and yeah, people are going to (watch it). **Participant 6***

A strong preference for use of pictures and storytelling to communicate health messages was also evident among the interview participants

*I think with pictures or, yeah, like storytelling might be a better option I believe. **Participant 10***

*I think the comic strips would be much better. **Participant 9***

#### Theme 4: preferred mode of intervention delivery

Most participants did not prefer the intervention to be delivered in a physical place.

*If you plan it (intervention delivery) for a place and everyone coming to that place and that may not be possible every time. **Participant 7***

Instead, participants stated that they would prefer the intervention to be digital and remotely delivered.

*Digital program is just more flexible and sometimes at work I don’t know what time I get off so it’s best for me to have that option open. **Participant 10***

### Codesign workshop (steps 4 and 5)

SA community members who met the inclusion criteria for the study including three members from the stakeholder group participated in the codesign workshop (*n* 12; Table 1). Most participants were male (*n* 8), were affiliated to the Hindu religion (*n* 8), completed tertiary education (*n* 9), were employed (*n* 8) and about half earned over 70 000 NZD (*n* 6). The mean age (s d) of the participants was 41 (s d 10·5) years, and most were established migrants who had lived in NZ for 10 or more years (*n* 8).

**Table 1:** Demographic characteristics of the codesign workshop participants (*n* 12)

Demographic characteristics	*n*	%
Mean age	41 years	
sd	sd 10·5	
*Sex*		
Male	8	67
Female	4	33
*Country of origin*		
India	8	67
Pakistan	1	8
Sri Lanka	2	17
Bangladesh	1	8
*Religion*		
Hindu	8	66
Muslim	2	16
Christian	2	16
*Education*		
Completed tertiary	9	75
Some tertiary or completed secondary school	3	25
*Employment*		
Employed	7	58
Not employed	5	42
*Household income per year*		
$NZ20,000–50 000	3	25
More than $NZ70,000	6	50
Do not want to answer	3	25
*Duration of residence in NZ*		
Early migrant (≥ 5years)	4	33
Established migrant (< 5years)	8	67

In addition to obtaining consensus from the workshop participants on the intervention delivery mode and formats for delivering the health messages, participants also suggested the use of information from credible sources to support the health messages embedded in the education comics and videos.

The design features of the intervention identified through the workshop included (1) the name of the intervention, (2) branding of the intervention, (3) the main and supporting characters of the comics and (4) the setting and scenarios for the education comics.

SADAI, an acronym developed from the study name, South Asian Diet and Activity Intervention, was agreed to be suitable as the name of the intervention. It was also agreed to use Sadai as the family name of the main characters. Sadai is Sanskrit, which is the mother language of all SA languages mean *‘Always’*. Using Sadai as a surname was considered a strong design feature as it captures the essence of this health-promoting intervention which was ‘being always healthy’.

The Kea, a unique parrot (Psittacine) species endemic to NZ was chosen to brand the intervention. ‘Aayus’ (a derivative of Ayush) meaning *long-life* in Sanskrit was chosen as the name of the pet Kea to capture the intervention’s objective of promoting good health and a long life.

*We need some sort of a pet in the mix, because that’s another element that we can use to convey a lot of activities and messages. (Group 3)*

**Fig. 2:**
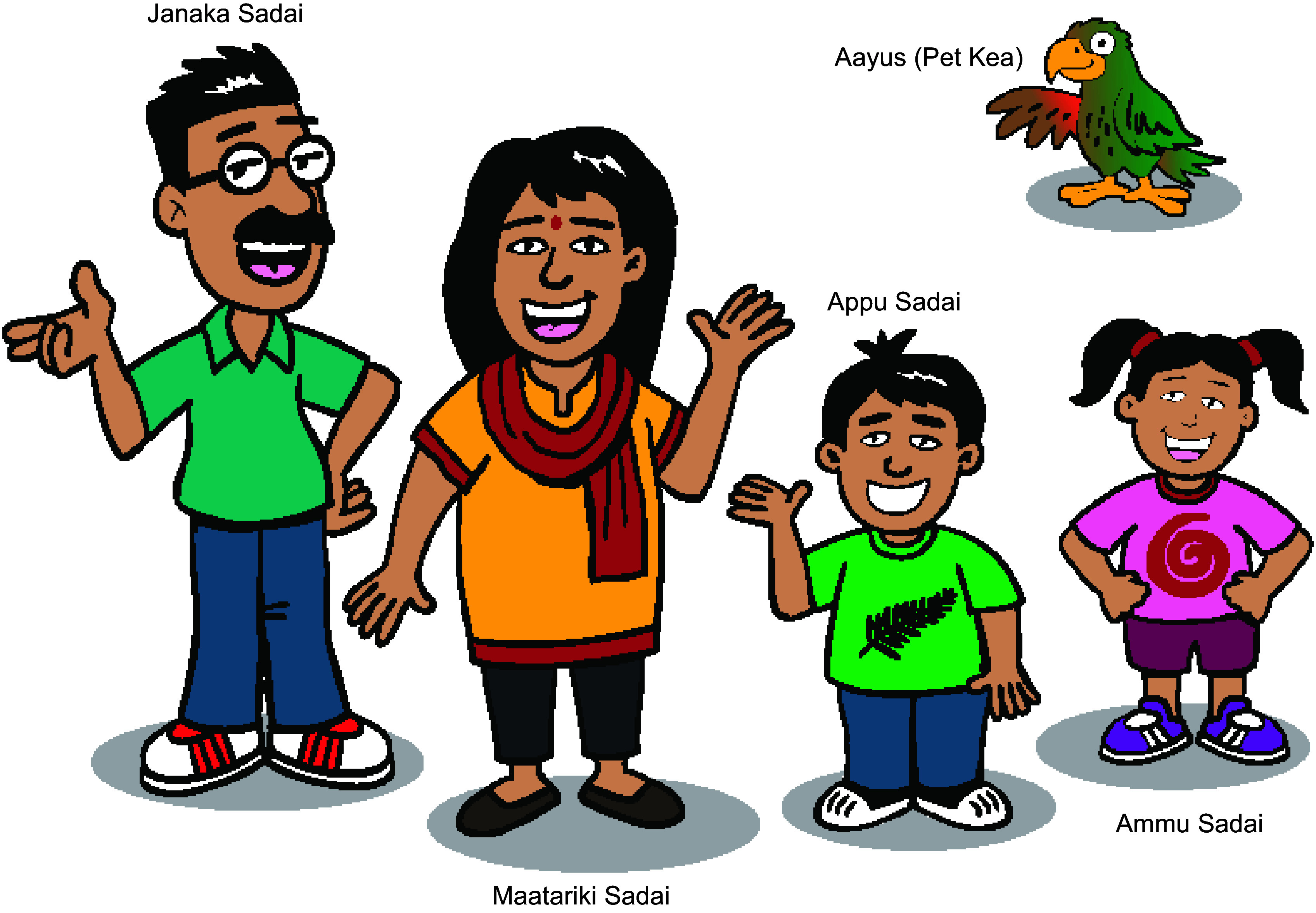
Main and brand characters of the education comics.

Figure 2 depicts the main and brand characters of the education comics. The characters for the education comics identified in this workshop were (1) dad, mum and children (2) friends and extended family, and (3) ethnic sports stars and superheroes. Branding the intervention as one specific for NZ SA was identified as a design feature of the intervention. Eight other characters were designed as extended family members (mother-in law) and friends (work colleague, neighbour, single men from home country who are flatmates and a sports star) of the Sadai family.

Family and social settings and scenarios, such as conversations between family members and friends – for example, dinner table discussion about food choices and food portions – were identified as the design feature of the education comics.

*. in one scenario like on the dining table, and the kids are playing in the background … the parents are eating deep fried foods like junk food, and then that they’re talking about how we need to change our food habits. (Group 1)*

*.in terms of the sports stars, like as South Asia have different countries, and everyone is fond of cricket. So having (people from) different countries, having group of people that chatting about being healthy. (Group 2)*

Incorporating the design features, settings, scenarios and characters from the codesign workshop, eleven digital education comics embedded with health messages addressing the target behaviours and knowledge gaps were developed (two examples included as see online supplementary material, Supplementary Files). The ratings of these comics by community volunteers were towards the higher end of the Likert scale for both cultural appropriateness (culturally appropriate/very appropriate) of and ease of understanding the embedded health message (easy/very easy to understand).

The second objective of the workshop was to identify volunteers with expertise from the SA community to be involved in coproducing the videos. Twelve volunteers were identified; however, due to the impact of COVID in 2020, only eight video clips were coproduced (Table 2). The remaining twelve planned video clips were produced as audio clips by SMP. The average duration of the video clips and audio clips were 6·40 and 3·60 min respectively.

**Table 2:** SADAI intervention components and formats

Modules	Comics	Video clips	Audio clips	Scientific snippets and other information
1. Improve awareness of the benefits of a healthy diet and regular physical activity	1. Why do we need to eat healthy foods and be active even if we are young to be healthy throughout life?	1. Too cold to exercise: Tips by a stakeholder via story telling on how to overcome the cold weather and be active	1 We are what we eat and do. 2 Balanced diets *v*. fad diets.	1. Benefits of healthy eating and physical activity 2. Can unbalanced diets cause disease? 3. What is the relationship between food and how our bodies function? 4. Relationship between food and mood 5. How does good diet and nutrition impact mental wellbeing
2. Improve awareness of a healthy breakfast, portion sizes and meal timing on health	1. Importance of regular breakfast and tips for easy cooking of traditional breakfast items 2. Importance of meal timings 3. Understanding portion size	1. Reading nutrition labels and understanding which ready-to-eat breakfast cereals are healthier: informational video	1. Understanding nutrition labels: health star rating. 2. Importance of meal timings. 3. Understanding recommended portion sizes of food groups. 4. Understanding the importance of regular and healthy breakfast.	1. What is a balanced diet? 2. Health star rating: How does this look on the packaged product.
3. Why and how to increase fruit, vegetables and lentil consumption	1. Nutritional value of and easy recipes using canned lentils 2. Nutritional value of and process of freezing vegetables	1. Growing herbs and salad vegetables in a small space using rice bags and yogurt pottles: gardening demonstration by a stakeholder. 2. Using locally available vegetables in South Asian cuisine: two cooking demonstrations by a stakeholder	1. Importance of adequate consumption of fruit, vegetables and lentils.	1. Nutritional quality of beans and lentils. 2. Why do we need to include legumes in our diet? 3. Are canned beans and lentils healthy? 4. Nutrition quality of fresh *v*. frozen vegetables. 5. Seasonal availability of fruit and vegetables in NZ.
4. Why and how to decrease dairy fats and oil consumption	1. How much butter, ghee and cheese are good for you? 2. How much oil is too much?	1. Low-fat yogurt options: informational video	1. Tips for using less oil in cooking.	1. Types of fat 2. Fat recommendations
5. Why and how to decrease high fat, sugar and salty snacks	1. Healthy takeaway options 2. Why we should limit high fat, sugar and salty snacks	1. Low fat, low salt quick and easy spicy homemade snacks: cooking demonstration by a stakeholder.	1. Healthiness of comfort foods. 2. Healthiness of takeaway foods.	1. Salt recommendations 2. Recommendations on takeaway and fast foods 3. What is snacking: Healthy snack options
6. Why and how to increase physical activity levels	1. Using active transport as a means of exercise for adults and children	1. Traditional dance as a means of exercise: warming up, exercise and cooling down using dance, demonstrated by a stakeholder.	1. Importance of active movement. 2. Household chores as exercise.	1. Physical activity recommendations 2. Tips to increase physical activity. 3. Links to locality-based walking tracks in NZ

SADAI, South Asian Diet and Activity Intervention.

### South Asian Diet and Activity Intervention (SADAI)

**Fig. 3:**
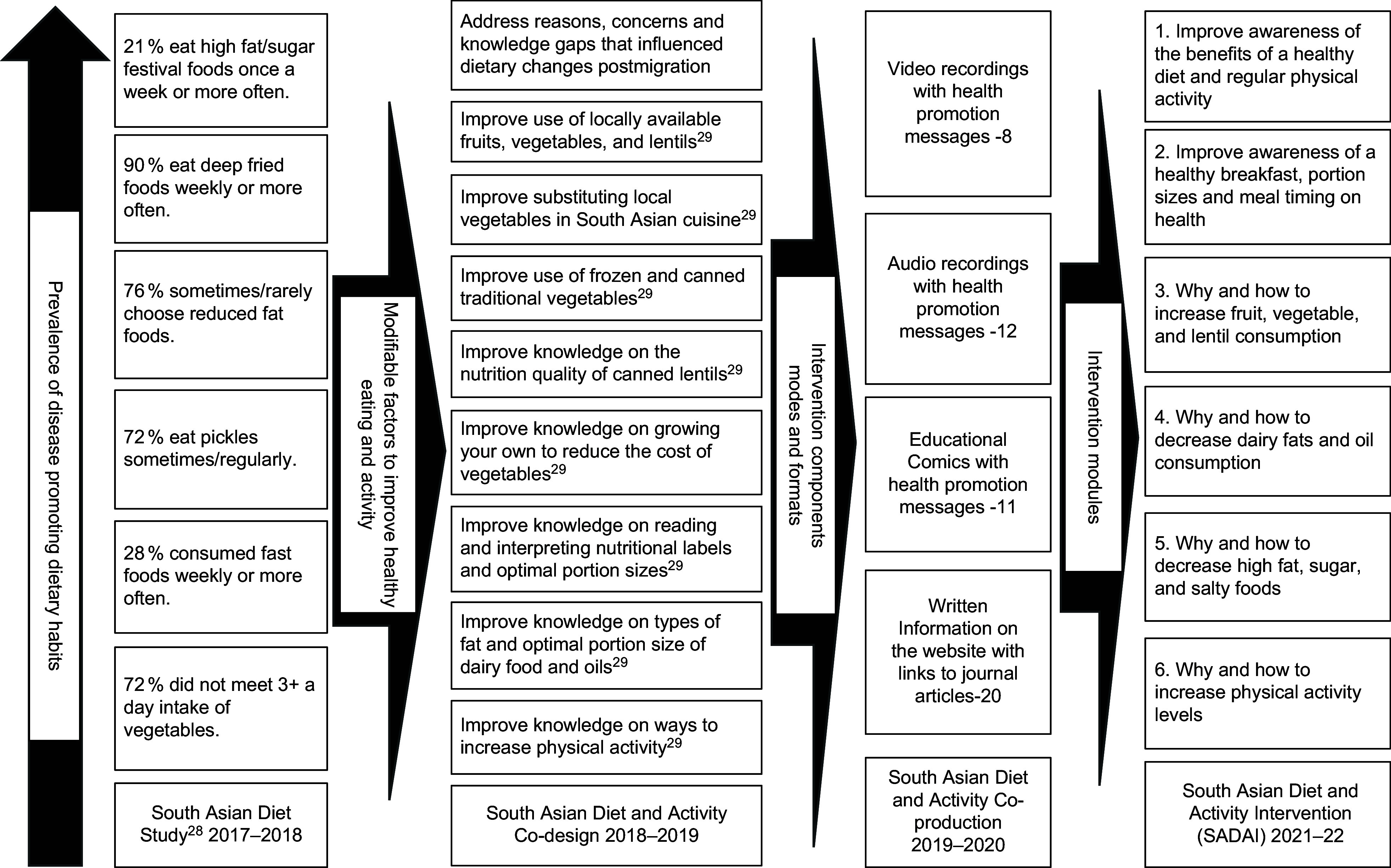
Process diagram of developing SADAI. SADAI, South Asian Diet and Activity Intervention.

The process and outcomes of the development of SADAI is illustrated in Fig. 3. In total, the intervention comprised of eleven education comics, eight video clips, twelve audio clips and eighteen scientific snippets. As shown in Table 2, the six intervention modules were (1) improve awareness of the benefits of a healthy diet and regular physical activity; (2) improve awareness of a healthy breakfast, portion sizes and meal timing on health; (3) provide information on why and how to increase fruit, vegetable and lentil consumption; (4) provide information on why and how to decrease dairy fats and oil consumption; (5) provide information on why and how to decrease high fat, sugar and salt consumption, and (6) provide information on why and how to increase activity levels.

## Discussion

The current study describes the process and outcomes of codesigning a health-promoting intervention to address disease-promoting dietary and activity behaviours. A PAR approach, more specifically, a codesign methodology, was chosen to enable going beyond just shared decision-making to shared ownership of the intervention by all involved. To the best of our knowledge, this is the first study to use codesign and coproduction to develop a health-promoting intervention to reduce the burden of diet-NCD among the SA diasporas. The digitised intervention comprised of six modules and health messages relevant to each module were developed as education comics, video clips, audio clips and scientific snippets.

The evidence supporting the positive impact of community engagement interventions on health behaviours is very strong^(33)^. The intervention development process in this study followed an iterative process inherent of codesign and was built on strong community partnership, engagement and involvement. The methodology adopted generated community-led solutions, enabling the identification of not just the components of the intervention, but also the format and modes of intervention delivery and coproducing the intervention.

Many initiatives have been undertaken to address the elevated risk of diet-related NCD within the SA diaspora^(16,17)^. One such intervention is the PODOSA trial in Scotland which was a family-based intervention comprising of four physical sessions with a dietician^(16)^. Such interventions are often not sustainable as they are not accessible beyond the life of the intervention without personal costs. As a result, they may show modest effects in the short term but not in the long term, as evidenced by the seven-year follow-up of the PODOSA trial^(18)^. The proportion of those who developed diabetes during the follow-up was similar in both groups (41 %; 32/79; *v*. 41 %; 38/85; hazard ratio of 0·86; 95 % CI 0·53, 1·38)^(18)^. In contrast, the Kaiser Permanente (KP) Northern California Heart Health for SA (HHSA) programme provided a 2-hour digitised educational class on risk factors for heart disease, culturally appropriate physical activity and dietary recommendations, stress reduction, cessation of substance abuse, with multiple accessibility^(34)^. The findings of the seven-year follow-up data highlighted the efficacy of this education programme in improving cardiovascular risk factors and reducing adverse cardiovascular events due to significantly lowered systolic and diastolic blood pressure, blood lipids and HbA1c^(34)^. Due to the digitised nature of the programme, the HHSA provided sustained and unlimited access to their educational programme in contrast to the PODOSA, which provided only time limited, face-to-face sessions with a dietitian. These findings highlight the higher impact of interventions with unlimited accessibility in achieving better health outcomes, supporting the preferred mode of intervention identified in the current codesigned study. Digitising the intervention also provides a pathway for scaling the intervention if proven effective for changing targeted behaviours. The codesign process of intervention development that we adopted enabled producing a health-promoting healthy diet and physical activity intervention with six modules, each addressing an identified dietary or activity behaviours that was disease-promoting among SA. The developed modules have the potential to facilitate delivering a targeted health-promoting digitised education programme to reduce the risk of diet NCD in SA immigrants.

A key output of this intervention development process was the eleven digitised education comics across the six modules. Education comics are informational in nature, and their role is less entertainment and more to transfer knowledge and communicate concepts in an engaging manner^(35)^. The education comics developed were rated highly by five SA volunteers for their clarity and cultural appropriateness. The codesign methodology provided the foundation of the comics. However, it is still necessary to evaluate whether the interpretation of the health message embedded in the developed comics by the target population aligns with the intended message. This will be achieved via pilot-testing the intervention, which is the next stage of this research. To our knowledge, no other health-promoting lifestyle intervention targeting SA have used this method of disseminating health messages.

As shown in Fig. 3, the majority of the intervention components can be traced back to the solutions generated by the participants. The thematic analysis reported in this manuscript identified the reasons for poor breakfast habits, one of which was a lack of time to prepare healthy traditional breakfast items. Although participants did not generate any specific solutions for this, addressing this issue was considered important and was included in the developed intervention (module 2: Comic 1; Table 2), especially as our previous study also indicated a shift from healthy breakfast habits to boxed sugary cereals^(29)^. Irregular meal timings were another issue for which participants did not generate any solutions. However, addressing this issue was considered important, especially given the prevalence of shift work among immigrants. Irregular meal timings, often associated with shift work, can have detrimental health consequences, such as obesity^(36)^ and metabolic syndrome^(37)^. Concerns such as long-term consequences of poor dietary habits leading to weight gain were also integrated into the intervention through the audio clips and scientific snippets in module 1 (Table 2).

The strength of this study lies in the strong community-centric approach, which enabled the development of an intervention designed and developed by the SA community. The use of codesign, guided by the theoretical framework of PAR, facilitated not only codesigning but also coproducing the intervention components. Three of the seven videos were produced by the community stakeholders. Several planned video productions were hampered by COVID-19 lockdown restrictions. Authors SMP and SYB are South Asians with extensive connections with the SA community in NZ of various faiths and socio-economic backgrounds. Hence, common limitations such as cultural sensitivity and language barriers did not hinder recruitment, data collection or analysis. Care was taken to include participants from the larger SA groups in NZ and of diverse religious affiliation. As the major SA group in NZ are Indians with an affiliation to Hinduism, the majority of our participants in the FGD, interviews and workshop were Indians who affiliated to Hinduism. Nevertheless, an inherent limitation of the current study is the generalisability of the findings to the broader SA communities in NZ and elsewhere, especially as the participants in the current study were highly educated, which was similar to that observed nationally^(38)^. The socio-economic profile of SA migrants in migrant receiving countries differ substantially. For example, NZ like the USA attracts highly educated SA via their skilled migrant category^(39)^. However, this is not the case in other migrant receiving countries, for example in Norway, where most migrants from South Asia have lower education levels^(40)^. This could be because of the difference in the migrant source countries, for example in Norway, SA are mainly from Sri Lanka and Pakistan and are mostly engaged in occupations that require lower qualifications^(41)^, whilst in NZ they are from India who are predominantly in jobs that require higher education levels^(42)^. Nevertheless, there is little difference in the prevalence of diet–NCD for example, diabetes among SA immigrants in other host countries including Norway and in NZ which is higher than the majority populations, primarily due to dietary transition^(31)^.

Overall, we believe that the intervention development process adopted resulted in an authentic codesigned intervention. However, the effectiveness of the intervention in changing dietary and physical activity-related disease-promoting behaviours must be investigated before implementation in the wider SA community of NZ, which is the next stage of this project.

### Implications for practice

This study highlights the feasibility of using a PAR approach to codesign and coproduce health interventions tailored to the SA community in NZ. In practice, health promotion programmes targeting diet-NCD should prioritise the involvement of community members in the design and production of the intervention. This approach not only ensures that the intervention resonates with the target population but also enhances its sustainability by fostering local ownership and engagement.

The preference for digital formats, such as educational comics and video clips, and the involvement of community experts in content creation are key factors for enhancing accessibility and communication. These findings can guide the development of future health interventions for other immigrant populations, ensuring they are accessible, engaging and culturally appropriate. Additionally, the incorporation of community stakeholders in content development can help ensure that health messages are effectively communicated and received.

## Conclusions

Using PAR approach to develop a health-promoting dietary and physical activity intervention to prevent diet-NCD in NZ SA is appropriate for codesigning and coproducing a comprehensive lifestyle health-promoting intervention. Future research should test the effectiveness of this codesigned intervention for improved health-promoting dietary and physical activity behaviours in the short term and reduced prevalence of diet NCD among the SA diaspora in the long term.

## Supporting information

Parackal et al. supplementary material 1 Parackal et al. supplementary material

Parackal et al. supplementary material 2 Parackal et al. supplementary material

## Supplementary material

For supplementary material accompanying this paper visit https://doi.org/10.1017/S1368980025100839
