# Supplementary figures and images for: Codesigning the South Asian Diet and Activity Intervention (SADAI): process and outcomes
Source: Public Health Nutr. 2025 Aug 27;28(1):e145. doi: 10.1017/S1368980025100839 (PMC12516638; doi:10.1017/S1368980025100839)

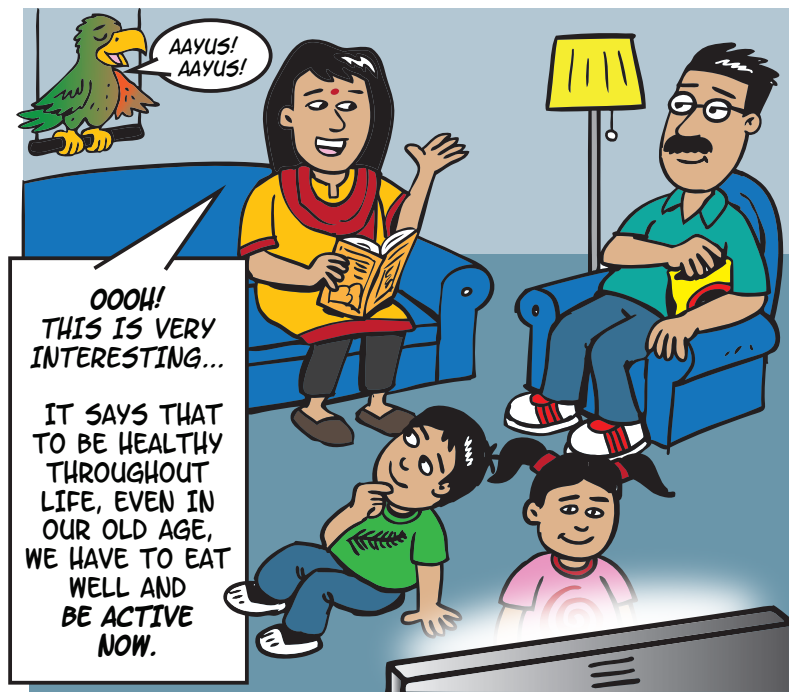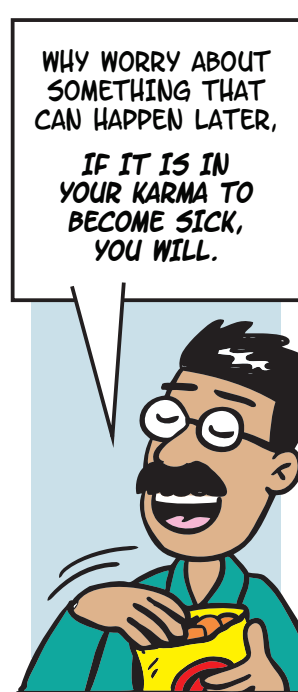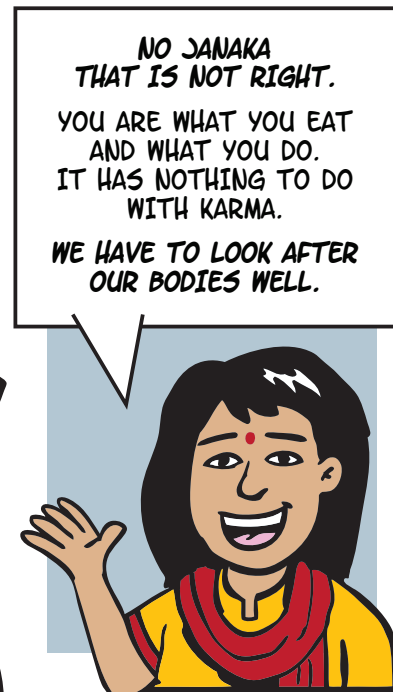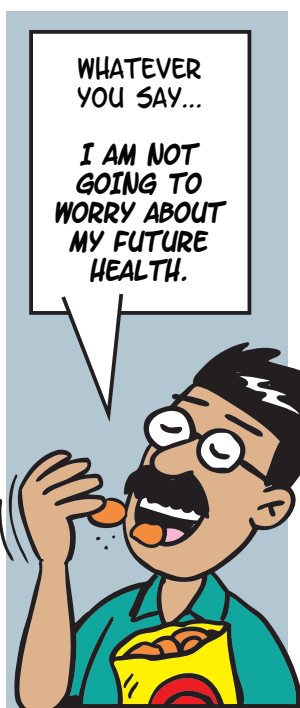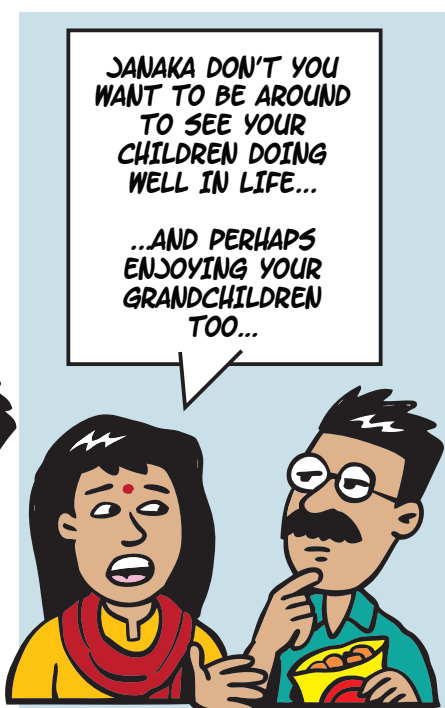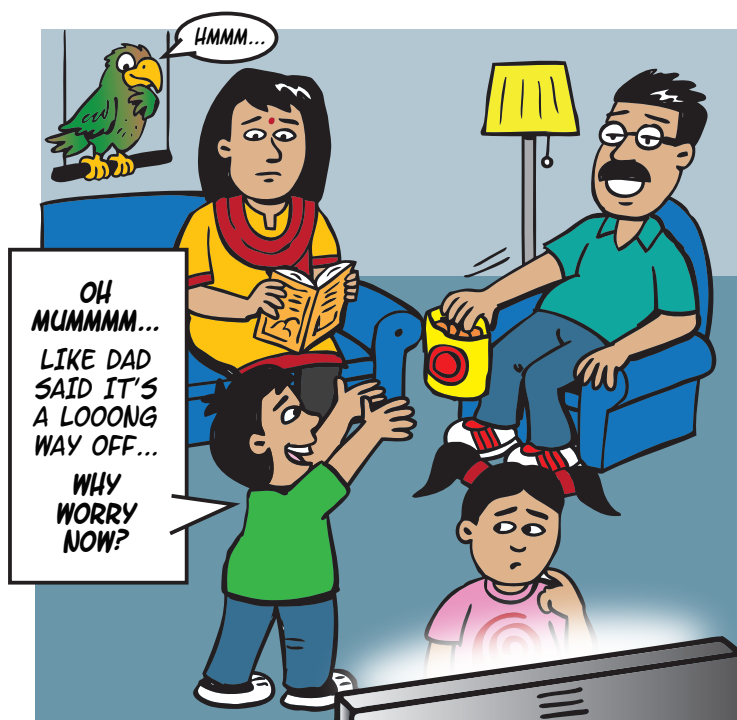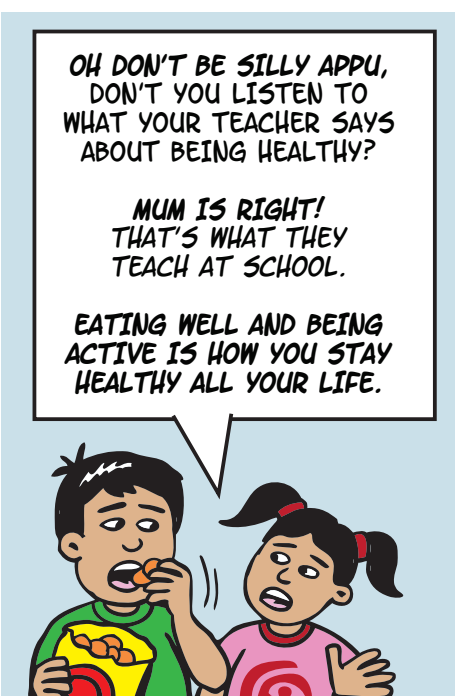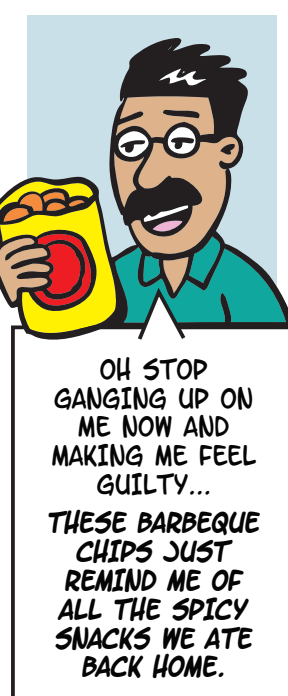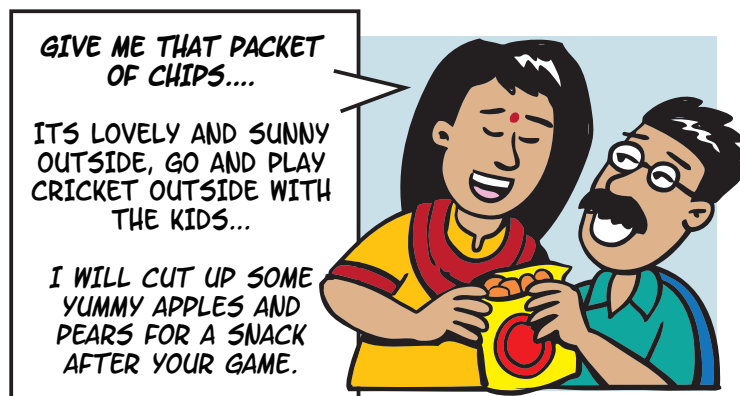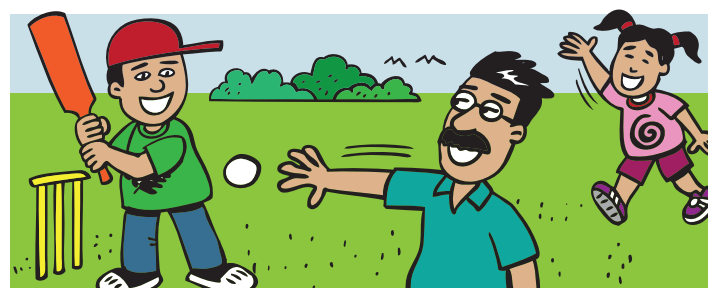

Supplement: Parackal et al. supplementary material 1 — Parackal et al. supplementary material [file S1368980025100839sup001.pdf]

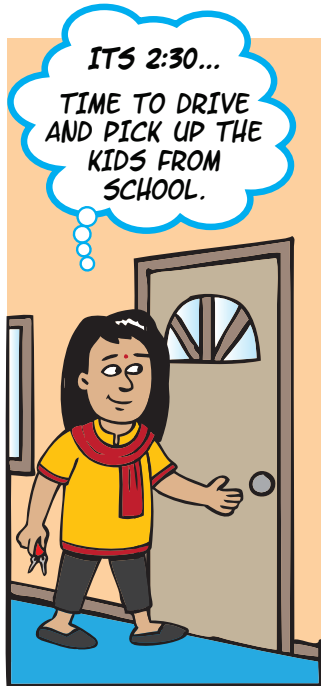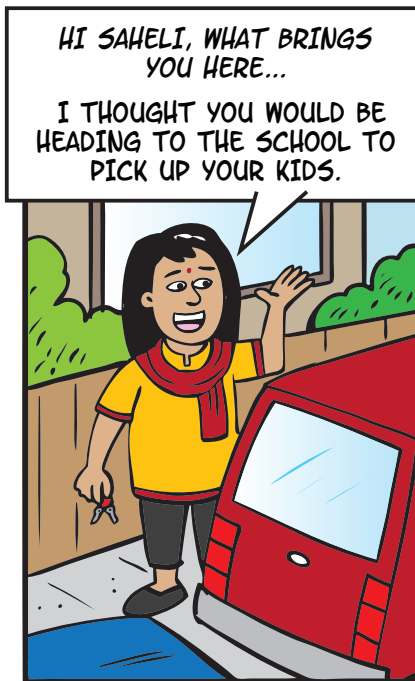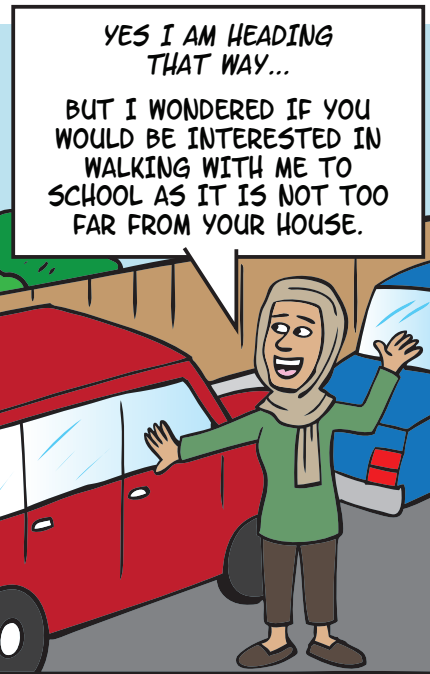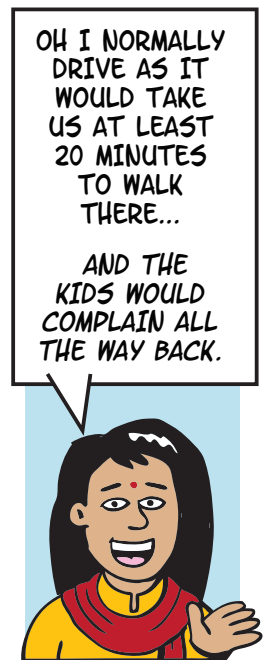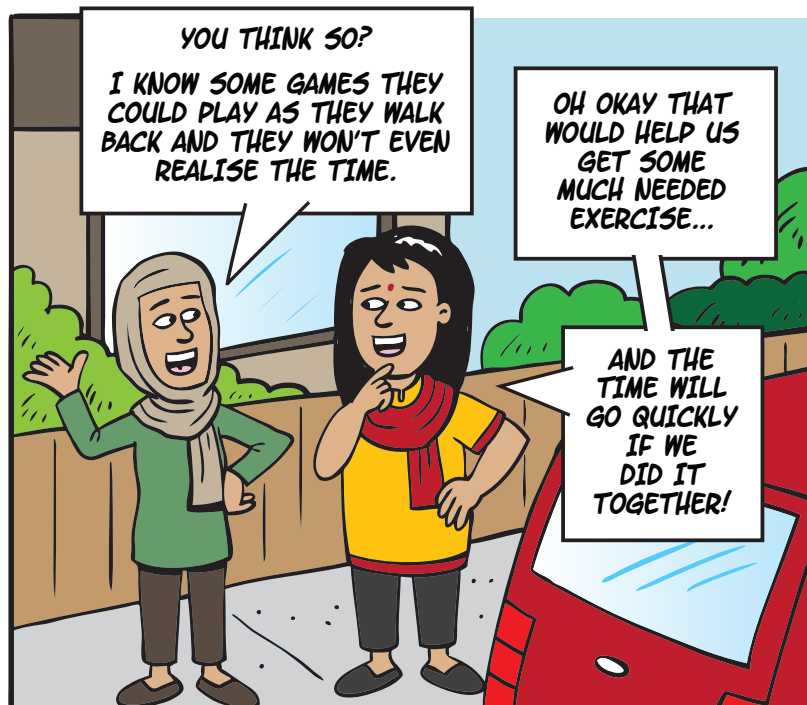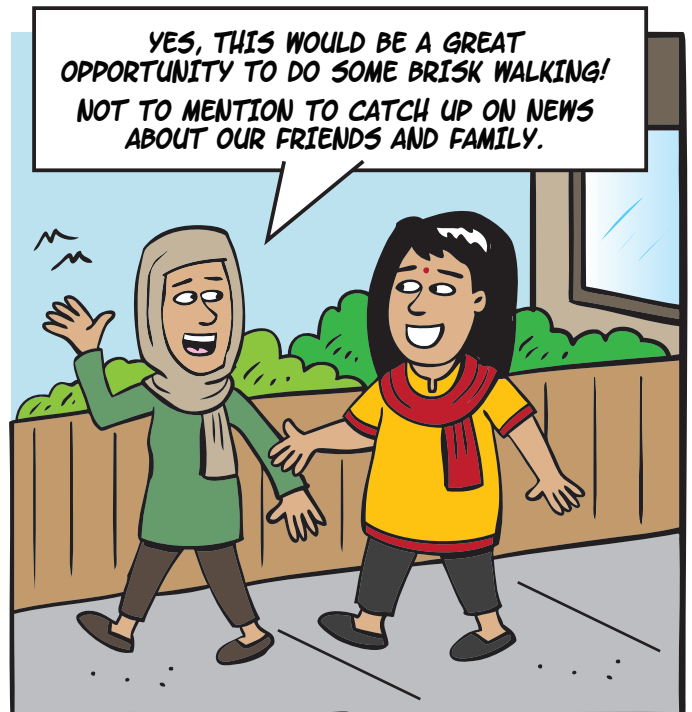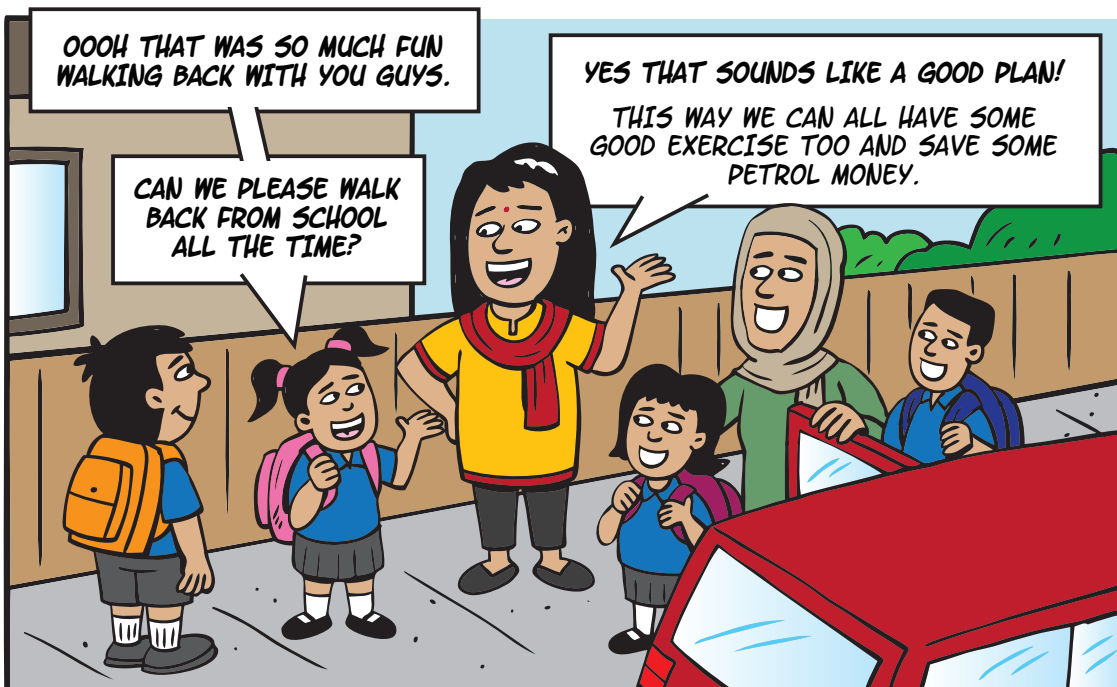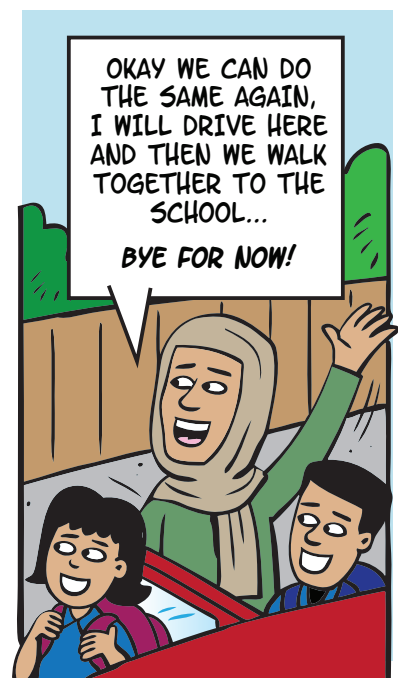

Supplement: Parackal et al. supplementary material 2 — Parackal et al. supplementary material [file S1368980025100839sup002.pdf]
